# The Response to Oxidative DNA Damage in Neurons: Mechanisms and Disease

**DOI:** 10.1155/2016/3619274

**Published:** 2016-01-31

**Authors:** Laura Narciso, Eleonora Parlanti, Mauro Racaniello, Valeria Simonelli, Alessio Cardinale, Daniela Merlo, Eugenia Dogliotti

**Affiliations:** ^1^Department of Environment and Health, Istituto Superiore di Sanità, Viale Regina Elena 299, 00161 Rome, Italy; ^2^Department of Food Safety and Veterinary Public Health, Istituto Superiore di Sanità, Viale Regina Elena 299, 00161 Rome, Italy; ^3^Department of Cell Biology and Neuroscience, Istituto Superiore di Sanità, Viale Regina Elena 299, 00161 Rome, Italy; ^4^IRCCS San Raffaele Pisana, 00166 Rome, Italy

## Abstract

There is a growing body of evidence indicating that the mechanisms that control genome stability are of key importance in the development and function of the nervous system. The major threat for neurons is oxidative DNA damage, which is repaired by the base excision repair (BER) pathway. Functional mutations of enzymes that are involved in the processing of single-strand breaks (SSB) that are generated during BER have been causally associated with syndromes that present important neurological alterations and cognitive decline. In this review, the plasticity of BER during neurogenesis and the importance of an efficient BER for correct brain function will be specifically addressed paying particular attention to the brain region and neuron-selectivity in SSB repair-associated neurological syndromes and age-related neurodegenerative diseases.

## 1. Introduction

Each cell in the human body receives tens of thousands of DNA lesions per day by a variety of sources. Therefore, cells have evolved a multifaceted response to counteract the potentially deleterious effects of DNA damage. The cellular response to DNA damage involves execution of DNA repair and activation of a repertoire of DNA damage signalling molecules (DNA damage response, DDR). The main DNA repair pathways, nucleotide excision repair (NER), base excision repair (BER), mismatch repair (MMR), homologous recombination (HR), and nonhomologous end-joining (NHEJ), are devoted to the repair of specific DNA alterations and complementary in some respects. NER is a multistep process that deals with damage causing significant distortion of DNA structure, such as UV-induced damage and bulky adducts (reviewed in [1]). BER corrects DNA from oxidation, deamination, and alkylation including single-strand breaks (SSB) which are all lesions that cause little distortion to the DNA helix structure (reviewed in [2]). MMR is an evolutionarily highly conserved repair pathway that corrects mismatches generated during DNA replication and escape proofreading (reviewed in [3]). Recombinational repair deals with the most lethal form of DNA damage, double strand breaks (DSB), by using an homologous DNA sequence as in the case of HR or requiring little or no sequence homology for efficient repair as in the case of NHEJ (reviewed in [4]).

The appropriate repair of DNA damage and resolution of replication problems is orchestrated by the DDR, through the action of sensors, transducers, and effectors that coordinate DNA repair with ongoing cell physiology. Signal transducers include ATM and ATM-Rad3-related (ATR) that are DNA damage-activated kinases that respond to different types of DNA lesions. Downstream of these proteins is two families of checkpoint kinases (Chk), the Chk1 and Chk2 kinases, that are targets of regulation by ATR and ATM kinases, respectively (reviewed in [5]).

DNA breaks arising from oxidative damage are a major threat for the genome stability of mature neurons [6]. This type of damage is mostly repaired by BER/SSBR. In this review, the plasticity of DNA repair during neurogenesis, the key role of BER/SSBR, and its brain region selectivity in neurological diseases will be specifically addressed by providing an update of recent findings. Moreover, original data on the characterization of the response to oxidative stress in neurons from different brain areas will be presented.

## 2. Plasticity of DNA Damage Type and Repair during Neurogenesis

**Figure 1:**
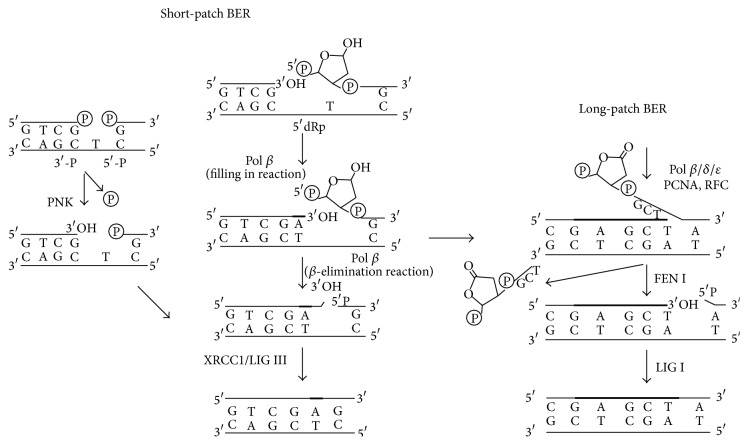
Simplified scheme for the short- and long-patch base excision repair pathways.

The regulatory networks of differentiation programs include genes that are involved in the response to DNA damage and cell death execution. As a consequence of this gene reprogramming, the mechanisms that deal with the maintenance of genome stability can change substantially in the transition from neurogenesis to nervous system maturation. By using* in vitro* cell differentiation systems, several studies have shown that DNA repair is downregulated during differentiation (reviewed in [7]). Indeed, the first evidence of differentiation-associated downregulation of DNA repair was provided by Hanawalt's laboratory in human hNT neurons [8]. In particular, when the repair of UV-induced DNA lesions was compared between terminally differentiated human hNT neurons and their precursor NT2 cells, it was clear that postmitotic neurons display attenuated global DNA repair but efficiently repair expressed genes (a pathway that was later called transcription-domain associated repair) [9]. Also, the mechanisms that control chromosome integrity, namely, telomerase and telomere-associated proteins, function as distinct telomere protection mechanisms during the processes of neurogenesis and neuronal maturation because of differentiation-associated transcriptional control. This impacts the response to DNA damaging agents as shown by the extreme sensitivity to telomere damage of newly generated neurons that are deficient in both the telomerase and the TRF2 telomere-binding protein [10]. The DNA damage response (DDR), the sophisticated cell network that monitors genome integrity, is also affected by differentiation-associated gene reprogramming. Carlessi et al. [11] showed that the differentiation of immortalized human neural stem cells* in vitro* is accompanied by an upregulation of ATM and the DNA-dependent kinase DNA-PK, sharp downregulation of ATR and Chk1, transient induction of p53, and the onset of apoptosis in a fraction of cells. The response to ionizing radiation (IR), including apoptosis, was dependent on ATM as shown by its attenuation following targeted silencing of ATM. Similarly, it was shown that DDR signalling and radiosensitivity were altered in terminally differentiated astrocytes as compared to their progenitors, neural stem cells (NSC) [12]. While NSC activated canonical DDR upon exposure to IR, astrocytes lacked functional DDR signalling with transcriptional repression of ATM leading to radioresistance. Astrocytes retained the expression of NHEJ genes and DNA-PK was shown to be the key player in the response to DNA damage. The efficiency of BER during neural differentiation was addressed by Sykora et al. [13]. They showed that terminally differentiated human SH-SY5Y neuroblastoma cells are more sensitive to oxidative damage than their undifferentiated counterparts. This is at least partially due to attenuated BER in postmitotic neurons that correlates with diminished protein levels of long-patch BER components (that are shared by DNA replication), such as flap endonuclease-1 (FEN1), proliferating cell nuclear antigen (PCNA), and DNA ligase 1 (Lig1) (Figure 1).

The evidence for the plasticity of DNA repair/DDR during cell differentiation, as inferred from these* in vitro* studies, is strengthened by* in vivo* studies in animal models where the effects of specific defects in DNA repair/DDR on neural development have been specifically investigated. During neural development, neural progenitors undergo symmetric divisions that expand the size of the progenitor pool before switching to an “asymmetric” mode of division wherein each round produces one progenitor cell and one “postmitotic” neuron. Newborn neurons then migrate from the proliferative zones to various CNS regions where they undergo further differentiation and become integrated into functional networks. In the early developmental stages, DNA repair plays a key role in the formation of a functional nervous system and the integrity of specific repair pathways is required along the developmental program [6]. This is well illustrated by mouse models with germline deletions of either Xrcc2 or DNA ligase 4 (Lig4), which are essential for the repair of DSB through HR and NHEJ, respectively. Xrcc2 ^−/−^ embryos display massive apoptosis in the brain by E10.5 when neural progenitor proliferation occurs, whereas no apoptotic cells are detectable in the brains of Lig4 ^−/−^ embryos until E12.5 when neural progenitors are differentiating into neurons [14]. Indeed in these different stages, cells are susceptible to different types of DNA damage. During proliferation, the most common type of damage is replication stress that is acted upon by HR and NHEJ. HR requires the presence of a sister chromatid and therefore this pathway is not available in neurons that have exited the cell cycle. In this cell type, NHEJ becomes the pathway responsible for DSB repair.

Challenging examples of the effect of the type of damage and its subsequent processing on the pathological outcome are two human syndromes, ataxia telangiectasia (A-T), a childhood neurodegenerative syndrome [15], and ATR-Seckel syndrome that presents severe neurodevelopmental defects [16]. These syndromes involve full or partial inactivation of the kinases ATM and ATR, respectively. These kinases respond to different types of damage that occur frequently during neural development: ATM to DSB while ATR is activated by RPA-coated single-stranded DNA, a lesion that may occur during replication fork collapse. Their lack of function leads to very different clinical outcomes: in the case of ATM to neurodegeneration and in the case of hypomorphic mutations of ATR to neurodevelopmental defects.

Oxidative DNA lesions, including SSB, are expected to be a frequent type of damage encountered by noncycling cells. The pathway of election for removal of oxidative DNA damage is BER. Targeted deletion of DNA polymerase *β* (Pol*β*), the main BER polymerase, causes neonatal lethality in mice [17]. Histological examination of the embryos showed extensive cell death in newly generated postmitotic neuronal cells in the developing central and peripheral nervous systems.

In conclusion, the plasticity of DNA repair/DDR during neurogenesis sets the tolerance to different types of DNA damage at different levels depending on the cell stage.

## 3. ROS: Sources and Role in Neurons

ROS and reactive nitrogen species (RNS) are generated by cellular metabolism and by exogenous agents. Metabolism-generated ROS can cause approximately 10.000 oxidative lesions per genome per day [18]. Neurons carry a high load of mitochondria. Almost 50 years ago, the first evidence was provided showing that the respiratory chain of mitochondria produces ROS [19]. The electrochemical gradient produced by the respiratory chain is used to synthesize ATP; however, some of these electrons inevitably leak out of the pathway leading to the production of O_2_^−∙^. These radical species can be very dangerous when produced in excess, but they are also important in redox signalling from the organelle to the rest of the cells. Controlled ROS generation is indeed necessary for optimal functioning of the CNS through fine-tuning of redox-sensitive signalling pathways. Brain mitochondria can also absorb large amounts of hydrogen peroxide when they utilize glycolysis as energy source [20]. Under the condition of neuronal cell damage, mitochondria are considered the main source of ROS during glutamate excitotoxicity [21]. ROS accumulation can damage neurons and mitochondria mediate both neuronal apoptosis and necrosis. Mitochondria act as platforms for the activation of caspases during apoptosis and participate in the dysregulation of Ca^2+^ homeostasis during necrosis. Another important source of ROS in damaged neurons are the nonmitochondrial nicotinamide adenine dinucleotide phosphate (NADPH) oxidases (NOX family) [22]. NOX enzymes are not only restricted to microglia but also expressed in neurons, astrocytes, and neurovascular system. There is now clear evidence for their role in various neurodegenerative diseases [23].

ROS-induced DNA damage has little, if any, specificity along the DNA strand. ROS-induced DNA damage includes base modification, deoxyribose modification, DNA cross-links, abasic sites, SSB, and DSB. SSB can occur directly by the disintegration of the oxidized sugar [24] or indirectly during BER of base damage as repair intermediates. SSB can also arise as a result of incorrect activity of DNA topoisomerase 1 (Top1) in which the enzyme remains covalently attached to the 3′ end of the break (Top1 cleavage complex) (reviewed in [25]). The 3′- and/or 5′-termini of most SSB must be restored to conventional 3′-hydroxyl and 5′-phosphate moieties to allow gap-filling and DNA ligation (Figure 1). Defective SSB repair (SSBR) can result in neurological diseases (see below). Although less frequently than SSB, DSB can also arise following replication past unrepaired SSB or when SSB encounter the transcription machinery or arise in close proximity. If not repaired they may have a dramatic impact on development.

## 4. BER/SSBR in Neuronal Cells

BER/SSBR is the main DNA repair mechanism in the removal of oxidized DNA bases and oxidized DNA break termini that are formed at high frequency in neuronal cells. BER proceeds through five steps: (i) base removal by a specific DNA glycosylase (DG); (ii) incision at the resulting abasic site by an AP-endonuclease (APE1); (iii) processing of the produced blocked termini of the gap; (iv) gap-filling by a DNA polymerase; and (v) resealing of the damaged DNA strand by a DNA ligase [26–28] (Figure 1). A brief biochemical characterization of the enzymes involved in these steps is provided below with special emphasis on their role in neurogenesis as inferred from* in vitro* and* in vivo* studies.

### 4.1. DNA Glycosylases: DNA Lesion Recognition and Removal

The first and most specific step of BER is the recognition of damaged DNA bases by distinct DGs. Eleven DGs have been identified in mammals and all of them recognize the specific DNA base by the same mode of action, that is, flipping base out of the DNA helix into an active site pocket [29]. Monofunctional DGs, such as uracil-DNA glycosylases (UDGs) and thymine DNA glycosylase (TDG), present only glycosylase activity and catalyze the base lesion excision mainly by hydrolyzing the N-glycosidic bond to generate an AP site [29]. The bifunctional DGs have an additional lyase activity and process the AP site via *β*  or  *β*/*δ* elimination reaction. They include the DGs specific for oxidized bases, such as 8-oxoGuanine (8-oxoG) DNA glycosylase (OGG1) and endonuclease VIII-like proteins [27].

UDGs are monomeric protein that recognize and excise uracil base from DNA [27]. Mammalian cells have five distinct UDGs: nuclear UNG2 is devoted to the repair of incorporated uracil (U:A base pair) whereas UNG1, SMUG1, TDG, and probably MBD4 all contribute to the repair of uracil in the U:G base pair (formed by the deamination of cytosine) [30]. Humans and mice have two different UNG isoforms, UNG1 and UNG2 localized in the mitochondria and in the nucleus, respectively [31]. The role of UNG1 in neurodegeneration is shown by the phenotype of conditional transgenic mice expressing a mutated version of UNG1 that present decreased mitochondrial respiration, apoptosis, neurodegeneration, and altered behaviour [32]. Feeding animals with a folate-deficient diet induced the degeneration of CA3 pyramidal neurons in Ung KO mice but not in wild-type animals [33]. Furthermore, folate depletion increased nuclear mutation rates in Ung KO mouse embryo fibroblasts (MEFs) [33] due to high levels of uracil in DNA [34] as a consequence of pool imbalance and/or cytosine deamination due to decreased levels of S-adenosylmethionine [35]. Depletion of UNG1 in cultured rat hippocampal neurons was also sufficient to induce DNA damage, upregulation of p53, and apoptosis [36].

Four different splice forms of OGG1 are present in mammalian cells, but only two of them are involved in the repair of 8-oxoG: OGG1-1a in nuclear DNA and OGG1-2a in mitochondrial DNA [37]. OGG1 initiates a canonical BER pathway that proceeds by the action of APE1, Pol*β*, and XRCC1/Lig3 to repair the damaged base [38, 39]. OGG1 is widely expressed and active in human and rodent brains [40]. Ogg1 KO mice have been used to examine the role of oxidative DNA damage in neuropathology. Liu et al. [41] reported that OGG1 protects neurons against cell death and its absence determines poorer functional outcome in mice under ischemic conditions. Aged Ogg1 KO mice showed a decreased spontaneous locomotor behaviour and a decrease in striatal dopamine levels [42]. During replication, the bypass of 8-oxoG by Pol*δ* determines the formation of the mismatch 8-oxoG:A that is recognized by MUTYH that mediates the removal of the adenine mispaired with 8-oxoG. Subsequently, Pol*λ* reconstitutes the correct 8-oxoG:C pair, thus allowing the intervention of OGG1 [43]. Both nuclear and mitochondrial isoforms of MUTYH are present in mammalian cells [44]. The analysis of single (Ogg1 KO or Mutyh KO) or double (Ogg1/Mutyh DKO) mutant mice revealed that Ogg1 KO mice exhibited severe striatal neurodegeneration, whereas mice lacking MUTYH or both OGG1 and MUTHY were resistant to neurodegeneration under the condition of oxidative stress [45]. These findings clearly indicate that 8-oxoG accumulation in neurons and microglia leads to neurodegeneration and suggest that the lack of MUTYH may protect the brain from oxidative stress by preventing SSB accumulation.

The mammalian homologs of the* Escherichia coli* endonuclease VIII, encoded by the Nei gene, are termed Nei-like (NEIL) 1, Nei-like 2, and Nei-like 3. NEIL1 and NEIL2 recognize oxidized pyrimidines such as thymine glycol, 5-hydroxycytosine, dihydrothymine, dihydrouracyl, and 5-hydroxyuracyl [46]. NEIL1 interacts with replication proteins and is preferentially involved in replication-coordinated BER [28]. NEIL2 seems to have a crucial role in the repair of oxidized bases in active genes (transcription-coupled BER, TC-BER) as suggested by its interaction with RNA polymerase II, TFIIH, CSB, and LIG3 both* in vitro* and* in vivo* [47, 48]. Neil2 KO mice indeed accumulate oxidative DNA damage mostly in transcribed regions of their genome [48]. TC-BER has been suggested also for the repair of 8-oxoG [49], requiring the involvement not only of OGG1 and RNA Pol II but also of NER factors such as XPA, CSB, and UVSSA, indicating the need for strict control of oxidative damage in active genes. NEIL3 recognizes and excises oxidation-induced hydantoin lesions [50]. Neural stem/progenitor cells from adult Neil3 KO mice are impaired in proliferation and hippocampal neurons present synaptic irregularities. Moreover, Neil3 KO mice are affected by learning and memory deficits, demonstrating that NEIL3 is pivotal for maintaining adult neurogenesis [51, 52].

The alkyladenine DNA glycosylase (AAG) mediates alkylation-induced tissue damage and whole-animal lethality [53]. In transgenic mice overexpressing AAG (Aag-Tg mice), alkylating agents induce extreme cerebellar toxicity and dramatically impaired motor function. Interestingly, these effects are prevented in Aag KO mice [54, 55] suggesting that AAG activity, in the presence of alkylation damage, determines an accumulation of toxic BER intermediates, while loss of AAG prevents their formation and promotes cell survival [54].

### 4.2. AP-Endonuclease 1: The Processing of Abasic Sites

APE1 is a multifunctional protein that has a central role in BER by processing the AP sites and in transcriptional regulation by redox activation of transcription factors [56]. APE1 cleaves the DNA sugar-phosphate backbone at a position immediately 5′ of AP sites to prime DNA repair synthesis but it can also correct oxidized abasic sites (reviewed in [57]). Ape1 knockdown in cortical neurons induced the accumulation of oxidative DNA damage after glutamate treatment, suggesting that APE1 has a pivotal role in the repair of oxidative DNA damage in neurons [58]. On the other hand, overexpression of APE1 is neuroprotective in neurons exposed to cisplatin [59] or hydrogen peroxide [60]. Moreover, the DNA repair function of APE1 protects differentiated neuroblastoma cells from apoptosis induced by hydrogen peroxide [61]. APE1 interacts with CDK5, which in turn phosphorylates APE1 at Thr 232, thus reducing its endonuclease activity and resulting in the accumulation of DNA damage in cortical neurons and in neuronal death after treatment with the neurotoxin 1-methyl-4-phenylpyridinium (MPP+) [62].

Besides APE1, the processing of SSB with 3′ and/or 5′ blocked termini generated by ROS involves end-processing factors that are instrumental in completing repair [27]. Human syndromes with varied neuropathology have been causally associated with defects in these factors. Their function and role will be addressed in the section on the role of BER/SSBR in human pathology (see below).

### 4.3. XRCC1: The Key Player in the Coordination of BER/SSBR

The scaffold protein XRCC1 orchestrates the coordination of the whole BER/SSBR process [26, 63]. XRCC1 interacts with several enzymes involved in BER/SSBR such as Lig3 [64], APE1, polynucleotide kinase/phosphatase PNKP, FEN1 [65], and DNA glycosylase OGG1 [66]. Moreover, XRCC1 interacts with PCNA and this interaction plays a central role in the DNA repair during DNA replication [67]. Xrcc1 KO mice die early in embryogenesis, indicating an essential role of XRCC1 in development [68]. Cerebellar granule cells from Xrcc1 heterozygous mice and Xrcc1 knockdown in human neuroblastoma cells show an accumulation of SSB and reduced survival following oxidative stress [69]. Neural-specific inactivation of Xrcc1 in mice induces loss of cerebellar interneurons, which causes a strong neuropathology. Moreover, loss of XRCC1 leads to the accumulation of SSB in the whole nervous system and abnormal hippocampal function [70].

### 4.4. The Resynthesis and Ligation Steps

Once damaged termini at SSB are restored to their conventional hydroxyl configuration, gap-filling and ligation will continue either via short-patch (SP) or long-patch (LP) BER, with distinct repair patches: one nucleotide in SP-BER and two or more nucleotides in LP-BER [65] (Figure 1). Pol*β* is involved in the resynthesis step in SP-BER while in LP-BER it incorporates the first nucleotide and Pol*δ* and Pol*ε* [71, 72] are possibly involved in the elongation step. PCNA participates in LP-BER, but a PCNA-independent and Pol*β*-dependent LP-BER has also been reported [73, 74]. In the SP-BER, after the dNMP insertion, the deoxyribose-phosphate (dRP) is removed by the dRP-lyase activity of Pol*β* [75] and the repair is completed by ligation by the Lig3/XRCC1 complex. In LP-BER, the dRP is displaced and Pol*β*/Pol*δ* polymerize tracts of DNA longer than one base [71, 76]. The strand displacement produces a flapped substrate that is refractory to ligation; this structure is recognized and excised by FEN1 [77, 78], followed by ligation by DNA ligase 1 (Lig1) [79]. Disruption of the coordination between Pol*β* and FEN1 in the processing of the flap structure leads to CAG repeat expansion that results in mutant Huntingtin protein expression in Huntington's disease (HD) [80, 81].

The choice of the BER subpathway is determined by multiple factors such as the type of lesion and the DNA glycosylase involved in its removal [38, 72, 82], protein-protein interaction, cell-cycle phase [65, 83], and/or differentiation status [13, 84]. As mentioned above, Sykora et al. [13] showed that differentiated human neuroblastoma cells are more sensitive to oxidative damage and present lower levels of proteins involved in LP-BER, such as FEN1, PCNA, and Lig1, thus relying mostly on Pol*β*-dependent BER for protection from endogenous damage. More recently, it has been shown that 50% Pol*β* reduction in a mouse model of Alzheimer accelerates synaptic and cognitive deficits determined by impaired autophagy and neurodegeneration [85].

## 5. Defects in SSBR in Neurological Diseases

PNKP is a DNA repair factor exhibiting both 5′-DNA kinase and 3′-phosphatase activities. Its capacity to remove 3′-phosphate generates 3′-OH termini, thus rendering DNA termini accessible for polymerases after base excision by the bifunctional glycosylases NEIL1 or NEIL2 [27]. PNKP interacts not only with the scaffold protein XRCC1 [86, 87] but also with XRCC4, a factor involved in NHEJ for the repair of DSB [88–91]. Mutations in PNKP have been recently identified as the cause of microcephaly with seizures (MCSZ), a syndrome characterized by profound neurodevelopmental microcephaly and early-onset seizures [92–94]. PNKP inactivation in murine neural progenitors induced neurodevelopmental abnormalities and postnatal death. In mice, in which a tamoxifen-inducible promoter was used to inactivate Pnkp after neurogenesis in different neural compartments, specific neural populations, including oligodendrocytes, were affected. These findings indicate that PNKP is required not only for neurogenesis but also for genome maintenance in mature neuronal cells, involving both BER and NHEJ [95].

Aprataxin (APTX) removes AMP from the 5′-termini of DNA breaks resulting from abortive DNA ligation events [96–98]. Loss of APTX in neuronal cells induces a defect in SSBR [98] and sensitivity to genotoxic agents [99]. Patients with loss of functional APTX are affected by ataxia with oculomotor apraxia-1 (AOA1), with progressive cerebellar ataxia [100, 101].

Tyrosyl-DNA phosphodiesterase 1 (TDP1) removes trapped topoisomerase peptides from 3′-termini of DNA breaks resulting from abortive topoisomerase 1 activity [102] and is involved also in the cleaning of others 3′-modified termini [103, 104]. Tdp1 KO mice show late onset progressive atrophy in the cerebellum and patients with loss of TDP1 are affected by spinocerebellar ataxia with axonal neuropathy (SCAN1) [105].

## 6. BER in Age-Related Neurodegenerative Diseases

Defective DNA repair has also been associated with age-related neurodegenerative disorders [106–109] such as Alzheimer's disease (AD) and Parkinson's disease (PD); however, the specific contribution of DNA damage to the etiology of these disorders has yet to be determined.

Parkinson's disease neurons present oxidative stress and genomic instability [110]. Dopaminergic neurons in the* substantia nigra* (SN)* pars compacta* of PD brains have high levels of mitochondrial DNA deletions, possibly related to respiratory chain deficiency [111]. In addition, an upregulation of the mitochondrial isoforms of the DNA glycosylases MUTYH and OGG1 was found in SN of PD patients [112, 113]. A significant proportion of dopaminergic neurons from PD patients was positive for phosphorylated APE1, while the proportion of dopaminergic neurons positive for total APE1 is similar in PD patients and normal individuals [62].

High levels of DNA breaks have been reported in neurons from AD patients [110]. Moreover, neurons from AD patients accumulate oxidized DNA bases both in nuclear and mitochondrial DNA [110]. Wang et al. have shown high levels of the oxidized DNA bases 8-oxoG in both nuclear and mitochondrial DNA from brains of Mild Cognitive Impairment (MCI) patients, the phase between normal aging and early dementia [114]. This finding suggests that oxidation of DNA bases is an early event in AD pathology and may play a role in neurodegeneration. Analysis of peripheral leukocytes derived from AD and MCI patients revealed increased levels of ROS-induced DNA damage [115]. Furthermore, treatment of human primary fibroblasts with oxidizing agents induces a gene expression pattern typical of fibroblasts from AD patients [116].

An alteration in gene expression of DNA repair genes has been observed in AD by several authors. A decrease of OGG1 activity has been described in brains from AD patients compared to healthy individuals [117]. Lower levels of UDG are also present in brains from AD patients compared to healthy controls [118]. MutT Homolog 1 (MTH1), which is critical to avoid the incorporation of oxidized DNA bases in nuclear DNA during replication, is downregulated in the hippocampus from AD patients compared to controls [119]. In addition, AD tissues have decreased levels of Pol*β* [120]. Huang et al. have shown that APE1 levels are similar in AD and healthy individuals, but AD brains have higher levels of phosphorylated APE1 [62].

An impairment of BER function has been described in sporadic AD patients: both UDG activity and Pol*β* activity are decreased in cell extracts from AD brain tissues. BER impairment is also present in MCI brains, where it correlates with the severity of the disease [118]. Further, both affected and nonaffected brain regions have a diminished BER activity, suggesting that BER dysfunction is a general feature of AD brains that could occur at the earliest stages of the disease and be pivotal in the progression of AD [118]. A study of BER capacity in mitochondrial extracts from AD brains shows that 5-hydroxyuracyl incision and DNA ligase activity are lower in AD brains [121]. A recent study [122] has evaluated markers of oxidative DNA damage, DNA repair, and cell cycle in hippocampus from three groups: (i) clinical-pathological AD, with AD neuropathology and clinical dementia (CP-AD), (ii) pathological AD, with neuropathology without clinical dementia (P-AD), and (iii) normal aging. Oxidative DNA damage was high in all groups, but subjects with CP-AD present reductions of DNA repair and cell-cycle inhibition markers and increases in cell-cycle progression and cell death markers when compared to both P-AD and normal subjects. No differences in all the markers were present between P-AD patients and normal subjects. These results indicate that cognitive decline may be associated with DNA repair impairment and cell-cycle deregulation.

## 7. Brain Region Selectivity and Neuronal Vulnerability in Neurological Diseases

The brain is the most complex organ of the human body and is characterized by different regions having distinct and specific functions. This functional multiplicity results from the presence of several different cell populations. Investigation of DNA repair activities in the brain showed differential activity patterns related both to the specific DNA repair system and the brain region analysed. An additional element, which distinguishes the DNA repair systems operating in the human brain, is the subcellular compartment where they act. Analysis of BER in five mouse brain regions [123], namely, caudate nucleus, frontal cortex, hippocampus, cerebellum, and brain stem showed that the activities of three major DGs, OGG1, UDG, and NTH1 are higher in the nucleus with respect to mitochondria and that the cerebellum is the region having the highest levels of nuclear DG activity. In contrast, mitochondrial glycosylase activities showed a pronounced variation among the brain regions analysed, which manifested a general decline associated with age. Instead, nuclear glycosylase activities decline appears to be limited to the cerebellum [123]. Analysis of mRNA expression pattern of NEIL1, NEIL2, OGG1, and NTH1 glycosylases confirmed a wide distribution of BER enzymes both in human and rodent brain regions, except for NEIL3 whose expression was revealed in a few cell populations and at early stages of postnatal development [40]. Again, the cerebellum is one of the brain areas showing the highest levels of DG transcripts. The preferential activity/expression of BER in the cerebellum could be related to the marked vulnerability and susceptibility of this brain region to neurodegenerative events observed in different human neurodegenerative syndromes associated with BER/SSBR defects. For instance, AOA1 and SCAN1 are characterized by a marked cerebellar atrophy leading to progressive ataxia. Aptx, the gene mutated in AOA1, is widely expressed in the nervous system and it has been detected in cerebellum, basal ganglia, cerebral cortex, and spinal cord [100, 101]. For many neurological aspects, AOA1 resembles A-T but lacks typical extraneurological features such as immunodeficiency and cancer susceptibility [124]. As revealed in two autopsied cases, the pronounced atrophy of AOA1 patients cerebellum is caused by a severe loss of Purkinje cells [125, 126]. Degeneration of posterior columns, spinocerebellar tracts, and anterior horn cells of the spinal cord was also observed [125]. Apart from cerebellar ataxia, other prominent clinical phenotypes of AOA1 patients are axonal sensorimotor neuropathy, cognitive defects, and chorea. The presence of aprataxin in the caudate nucleus [100], the* in vivo* detection of caudate nucleus hypoperfusion [127], and a reduction of dopamine transporter density in caudate and putamen of AOA1 brains [128] suggested that aprataxin mutations in basal ganglia could affect the function of this brain region, thus leading to choreoathetosis. However, it should be noted that morphological alterations of basal ganglia have not been observed in AOA1 patients. Cognitive disturbances have also been found in AOA1 patients which are consistent with a possible disruption of the frontocerebellar pathways [127].

SCAN1 has a later onset compared to AOA1. In the human brain, Tdp1, the gene mutated in SCAN1, is highly expressed in different regions including the cerebellum (granule and Purkinje cells), dentate nucleus, spinal cord, and dorsal root ganglia [129], similar to mouse brain. Although there are not reported autopsy studies from SCAN1 patients, the brain expression profiles of TDP1 in nondiseased brains together with progressive ataxia and axonal sensorimotor neuropathy typical of SCAN1 individuals are consistent with the involvement of the TDP1 expressing regions in the pathogenesis of SCAN1. It is noteworthy that SCAN1 patients do not manifest cognitive defects as compared to AOA1 individuals [102].

Another gene involved both in nuclear and mitochondrial BER and associated with cerebellar dysfunction is Pnkp. Recently, the finding of severe cerebellar atrophy [93] in two Dutch siblings affected by MSCZ with a homozygous mutation in Pnkp and in 11 individuals of nine Portuguese families affected by early-onset recessive AOA [130] confirmed the cerebellum as one of the most vulnerable brain regions in DNA repair syndromes. As reported in AOA1 patients, beside ataxia, cognitive impairments were also observed [93, 130] in several individuals, leading even to severe dementia in some cases [130]. Although PNKP has been identified in normal and pathological human cerebellum [131], a detailed analysis of expression levels and/or activity of PNKP in human brain is not yet available.

A clear picture emerging from the analysis of these diseases is that the cerebellum appears to be the brain region with the highest vulnerability to defects in BER/SSBR activities. The high expression/activity levels of BER core proteins (OGG1, UDG, NTH1, NEIL1, and NEIL2) and end-processing DNA repair factors such as APTX and TDP1 in the cerebellum could be suggestive of a high susceptibility of this brain region to DNA lesions and especially to oxidative DNA damages. Consistently, it has been shown that cerebellar granule neurons and CA1 neurons are particularly vulnerable to oxidative stress stimuli compared to other neurons such as cortical and CA3 neurons [132, 133]. This feature correlates with the marked loss of cerebellar granule cells in aged individuals [134, 135] and the decline of BER glycosylase activities specifically detected in the cerebellum during aging [123]. Importantly, data derived from transcriptomic analyses on cerebellar granules and cortical neurons showed that some genes related to the DNA damage response and repair (such as Hmgb2 and Pold1) are markedly more active in the cerebellar neurons [136]. A parallel result was obtained on CA1 neurons as compared to CA3 neurons [136]. The finding that, under basal conditions, cerebellar granule neurons have 25% lower ATP levels with respect to cortical neurons could also account for the selective vulnerability of cerebellum to oxidative stress, a condition requiring a high energy demand to cope with DNA damages.

As described for SCAN1 and AOA1, clinical phenotypes characterizing age-related neurodegenerative diseases are associated with the specific brain regions and population of neurons targeted, even though important causality issues remain to be addressed [140]. In AD, early memory deficits are caused by the selective degeneration of pyramidal neurons in the entorhinal cortex, subiculum and CA1 of the hippocampus, and accumulation of *β*-amyloid in frontal, temporal, and parietal cortex. In particular, cholinergic neurons are the most affected neuronal type [141]. In PD, dopaminergic neurons of the SN are targeted [142] and their degeneration accounts for the major clinical manifestations of PD. Another neuronal type specifically targeted in neurodegenerative diseases is GABAergic neuron, whose loss characterizes both spinocerebellar ataxia-1 (SCA1) and Huntington's disease (HD), two polyglutamine diseases. It is noteworthy that the cell population affected in SCA1 is represented by giant Purkinje cells of the cerebellum [143], whereas spiny neurons of the striatum degenerate in HD [144]. Interestingly, the genes causing SCA1 and HD are expressed in both cerebellum and striatum; however, the reason why both regions are not affected in both diseases is not known.

Although there is no evidence for a region- and neuron-specific DNA damage response in neurodegenerative diseases, it is tempting to speculate that neurons that are selectively affected in these diseases could be both selectively vulnerable to specific DNA damage inducers (e.g., ROS) and/or less responsive to DNA repair.

To address this issue, we have compared the response to oxidative DNA damage of different types of neurons derived from mouse cortex, cerebellum, and hippocampus.

**Figure 2:**
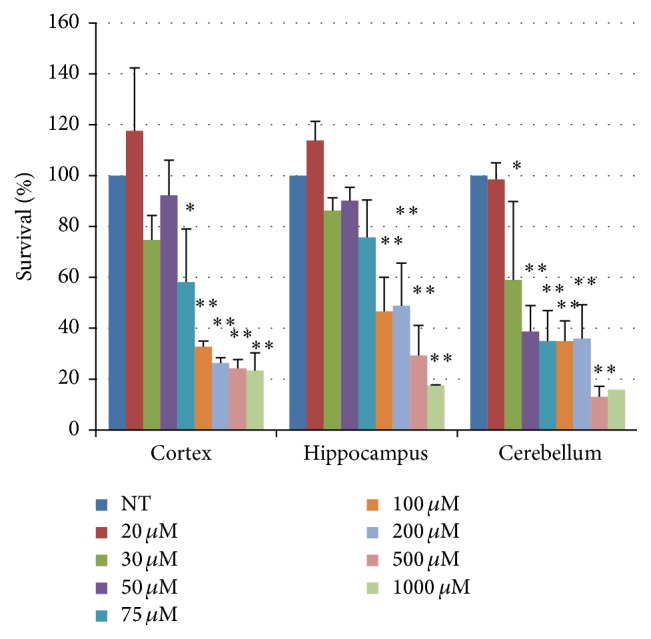
Cell survival as detected by the MTT assay in neurons from different brain regions treated with increasing H_2_O_2_ doses for 30 min. Error bars indicate the standard error. ^*∗*^ *p* < 0.05 and ^*∗∗*^ *p* < 0.01 versus untreated (NT) by Least Significant Difference (LSD) test. Two-way ANOVA did not show any significant difference in the response to treatment in the neurons derived from the three different brain regions (cell cultures were prepared as previously described) [137–139]. In more detail, C57BL wild-type mice were sacrificed at postnatal day 5 (P5) for cerebellar granule cell cultures, whereas at day 17 embryos were collected from pregnant mice for preparation of cortical and hippocampal neurons. Brain regions were dissected and dissociated and cultures were maintained in appropriate media.

**Figure 3:**
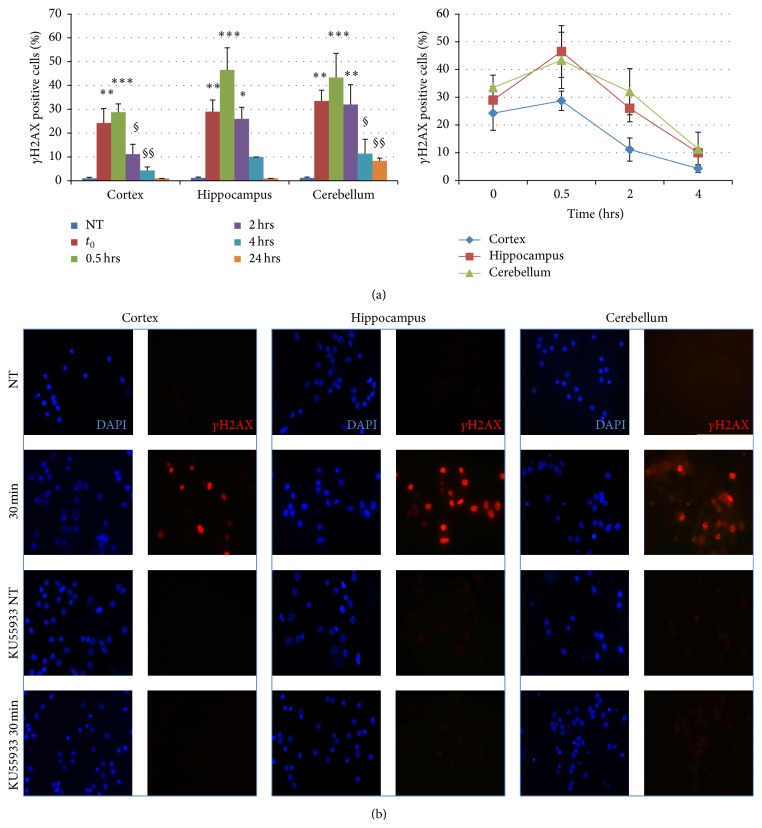
H_2_O_2_-induced DNA damage and repair as detected by *γ*H2AX foci formation in neurons derived from different brain regions. (a) Left: percentage of cells bearing 10 or more *γ*H2AX foci at different times after treatment with 20 *μ*M H_2_O_2_ for 30 min. One-way ANOVA and* post hoc* analysis Least Significant Differences (LSD) indicated a significant increase of *γ*H2AX positive cells immediately and 30 minutes after the treatment in all brain regions. Two-way ANOVA did not show any interaction between the treatment and the neuronal cell type. Right: kinetics of *γ*H2AX dephosphorylation following posttreatment times up to 24 hrs. ANOVA test shows any interaction between treatment and the three types of neurons on slopes of focus kinetics. For each time point, at least 100 nuclei were examined. Error bars indicate standard error. ^*∗*^ *p* < 0.05 versus untreated (NT) by LSD test; ^§^ *p* < 0.05 versus *t*_0_ by LSD test; ^*∗∗*^ *p* < 0.01 versus untreated (NT) by LSD test; ^§§^ *p* < 0.01 versus *t*_0_ by LSD test; ^*∗∗∗*^ *p* < 0.05 versus untreated (NT) by LSD test. (b) Immunofluorescence of *γ*H2AX (red, Ser 139; Millipore, Billerica, MA, USA) in neurons exposed to H_2_O_2_ (20 *μ*M, 30 min) with or without 1 hr pretreatment with a specific ATM kinase inhibitor KU55933 (10 *μ*M). Nuclei are stained with DAPI in blue. One representative experiment is shown.

**Figure 4:**
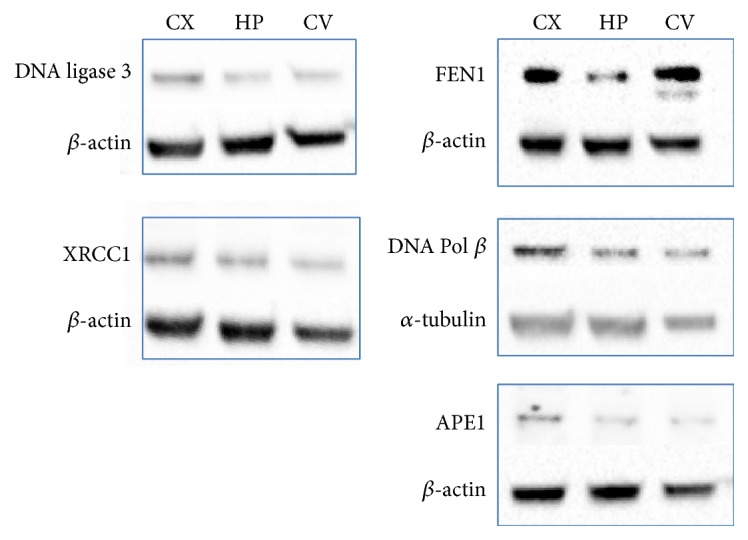
Levels of BER enzymes in neurons derived from cortex (CX), hippocampus (HP), and cerebellum (CV). Immunoblotting was carried out by using antibodies specific for DNA ligase 3 (103 kDa, BD Biosciences Pharmingen, San Diego, CA), FEN1 (43 kDa, Abcam, Atlanta, USA), XRCC1 (85 kDa, Bethyl Laboratories, Inc.), APE1 (35.5 kDa, Santa Cruz Biotechnology, Inc.), and DNA Pol*β* (38 kDa, Trevigen Inc., Gaithersburg, MD). *β*-actin (40 kDa, Sigma) and *α*-tubulin (55 kDa) were used as loading controls. Western blot analysis was conducted on 15 *μ*g of whole cell extracts. One representative experiment is shown.

All three types of neurons showed a significant H_2_O_2_ dose-related decrease in survival (Figure 2). Neurons from cerebellum seem to be more sensitive (LC_50_ = 29.8 *μ*M) to oxidative stress when compared to neurons from cortical and hippocampal regions (LC_50_ = 41.5 and 55.2 *μ*M, resp.). The efficiency of DDR was monitored by the formation and repair of *γ*H2AX foci. The number of foci-positive nuclei immediately after treatment was lower in cortical neurons as compared with neurons from cerebellum and hippocampus, suggesting a less effective DDR in cortex (Figure 3(a), left). During the posttreatment repair time, the percentage of *γ*H2AX foci-positive cells declined in all three types of neurons with the same rate (Figure 3(a), right) and at 24 hr posttreatment the repair was almost completed in all neuronal cell types. The presence of the specific ATM kinase inhibitor KU55933 fully abolished the appearance of *γ*H2AX foci (Figure 3(b)), indicating that this kinase is activated following H_2_O_2_-induced DNA damage in neurons from the three brain regions analysed. An ATM-dependent DDR has also been described following H_2_O_2_ treatment in postmitotic myotubes [145] strengthening the importance of this kinase in the signalling of DNA breaks [146]. Finally, when the protein levels of key BER enzymes, such as APE1, XRCC1, Pol*β*, Lig3, and FEN1, were measured (Figure 4), no significant differences were found by comparing neurons derived from the three different brain regions. A notable exception is FEN1 that was higher in neurons from the cerebellum and the cortex as compared with neurons from hippocampus. It has been shown that the expression levels of DNA replication/repair proteins, including FEN1, predict regional somatic repeat instability in the brain [147]. We confirm elevated expression of FEN1 in the cerebellum, thus supporting the hypothesis that in this brain region it may account for the reduced somatic instability as compared with other regions (e.g., striatum) [81]. Therefore, our data support the hypothesis that brain region differences in BER/SSBR activities and/or DDR efficiency may contribute to the brain region selectivity and neuronal vulnerability in neurological diseases.

## 8. Conclusions

There is emerging evidence for an important role of BER/SSBR in the control of genome stability in the nervous system. Indeed, a malfunction of almost all the key players of this pathway has been associated with neurological alterations. This finding confirms that neuronal cells need to be protected from oxidative damage that is mostly repaired by BER. Oxidative damage is produced at high levels in the brain due to high level of tissue oxygen consumption. SSB that are produced during BER are a major threat to mature brain as shown by the neurological consequences of defective SSBR. There is some evidence that the mechanisms that control DNA damage in neurons may vary depending on the brain region.

In SSBR-associated neurological syndromes, specific brain areas are affected, with the cerebellum being the most vulnerable one. In contrast, further investigation is required to determine if differences in response to DNA damage underlie the brain region selectivity observed in neurodegenerative diseases.

A better understanding of DNA repair/DDR mechanisms in the nervous system may open new avenues in the design of innovative therapies.
